# Social Relatedness and Physical Health Are More Strongly Related in Older Than Younger Adults: Findings from the Korean Adult Longitudinal Study

**DOI:** 10.3389/fpsyg.2018.00003

**Published:** 2018-01-19

**Authors:** Eunsoo Choi, Yuri Kwon, Minha Lee, Jongan Choi, Incheol Choi

**Affiliations:** ^1^Center for Happiness Studies, Seoul National University, Seoul, South Korea; ^2^Department of Psychology, Seoul National University, Seoul, South Korea

**Keywords:** loneliness, perceived social support, physical symptoms, age factors, chronic health conditions

## Abstract

Previous research indicates that social relatedness is beneficial to physical health; however, findings on the relative strength of the relationship between these variables have been inconsistent. The present study employed cross-sectional survey (Study 1) and a daily diary survey (Study 2) to examine the link between social relatedness and physical health by age. Using a representative sample of Korean adults (*N* = 371) aged from 20 to 69, Study 1 examines the link between social relatedness (loneliness, perceived social support) and physical health (physical symptoms, chronic health conditions) using age as a moderator. The results show that participants' age moderates the association between social relatedness and physical health. Study 2 (*N* = 384) further corroborated the findings from Study 1 by showing that when controlling for the physical symptoms experienced prior to the daily diary reports, the level of loneliness experienced over a 13-day period exacerbates the age differences in the physical symptoms. The present study thus provides converging evidence that social relatedness plays a significant role in physical health, particularly in the older population.

## Introduction

It has been well established that social resources constitute one of the major protective factors for both physical and mental health (Holt-Lunstad et al., 2010; Uchino et al., 2012). Social resources in terms of one's subjective evaluation of the availability of or access to social relationships, are conceptualized in a variety of ways. Loneliness, or the perception that one lacks social resources, is a pervasive experience in most modern societies (Victor and Yang, 2012; Victor and Sullivan, 2015). Loneliness is a fundamentally a distressing feeling that stems, in part, from a lack of fulfillment of one's need or desire to connect with others (Cacioppo et al., 2002). Compared to depression or anxiety, loneliness is a type of negative emotional experience that involves the social interdependence of individuals; thus, it is called a socially engaging (vs. disengaging) emotion (Kitayama et al., 2006; Uchida and Kitayama, 2009). The so-called socially engaging emotions are associated with people's happiness, particularly so in an interdependent cultural context such as in East Asian cultural context (Kitayama et al., 2006).

The counterpart of loneliness, perceived social support is the perception that one is cared for and that support from others is available (Caplan, 1974; Dunkel-Schetter and Bennett, 1990; Cohen, 1992, 2004). Though these two indices reflect the objective reality of social connectedness, as indicated by the number of close others or social contacts, they also refer to the subjective perception of social relatedness, which is primarily emotional in nature (Dunkel-Schetter and Bennett, 1990; Bolger and Amarel, 2007). Given the detrimental effects of negative emotional experiences on one's mental and physical state (Assari and Lankarani, 2016; Palumbo et al., 2017), it is no surprise that feelings of loneliness are associated with poor health.

Much empirical evidence, obtained from both longitudinal and cross-sectional studies, has shown that social relatedness has advantageous effects on physiological processes, ultimately positively affecting chronic diseases and longevity (see Holt-Lunstad et al., 2010 for a review; Hawkley et al., 2010). Previous research documents that loneliness predicts not only lower levels of psychological well-being (Cacioppo et al., 2010), but also lower self-rated health (Nummela et al., 2011), less physical activity (Hawkley et al., 2009), and worse physical functioning (Luo et al., 2012). While loneliness is the psychological measure of the lack of social relatedness, perceived social support—a natural counterpart of loneliness—is a measure of the positive presence of social relatedness. As such, perceived social support is found to be associated with better biological outcomes (Uchino et al., 1996; see reviews by Holt-Lunstad et al., 2010). A substantial amount of evidence links perceived social support with physical health status, including mortality rates (Brummett et al., 2001; Holt-Lunstad et al., 2010), immune functions (Baron et al., 1990; Kiecolt-Glaser et al., 2010), chronic health conditions such as cardiovascular disease (Barth et al., 2010), as well as self-reported health status (Alpass and Neville, 2003).

Despite the accumulating research on the effect of social relatedness on physical health, however, it is unclear whether the degree of the relationship between social relatedness and physical health is the same for individuals of different ages. Since chronic conditions and health problems are naturally more common in late adulthood, much of the research on the relationship between social relatedness and health has been biased toward older adults (Brooks et al., 2014; Yang et al., 2016) and there is limited evidence regarding the potentially varying degrees of associations between loneliness or perceived social support and physical health according to age (Umberson et al., 2010; Brooks et al., 2014). To address this limitation, the present research included participants of a broad age range (20–69) and examined whether and how the strength of the association between social relatedness and physical health varies with age.

### Social relatedness and health across age groups

There are reasons to expect that the link between social relatedness and physical health may vary by age; specifically, the association between social relatedness and physical health may be stronger for older adults than for younger adults. First, according to socioemotional selective theory, individuals begin to perceive that they have less time left as they age, which leads them to be more aware of their social relationships, especially close, intimate relationships (Carstensen, 1992; Carstensen et al., 1999). Although the number and size of social ties decrease as people grow older (Tornstam, 1997), intimate social relationships are maintained and may even be more salient to the well-being of older (vs. younger) adults (Matt and Dean, 1993; Umberson et al., 2006; Elliot et al., 2017). By the time individuals reach older adulthood, their relationships have accumulated certain benefits that are possible in close relationships that have lasted a long time (Chopik, 2017). Second, it has long been suggested that social relatedness has a buffering effect on well-being in stressful situations (Cobb, 1976; Gore, 1981). Given that getting old is inevitably associated with a range of life stressors, including deterioration in physical health (Kanis et al., 2000; Walther et al., 2017); challenges in financial circumstances (Kahn and Pearlin, 2006; Wilkinson, 2016), and stressors in social relations such as the death of a spouse or friend (Steptoe et al., 2013), social resources have a particularly significant role in adaptive functioning in later life stages.

Recently, researchers have started to pay attention to the lifespan approach to examine the associations between social relatedness and physical functioning in different life stages and for extended periods of the life course (Brooks et al., 2014; Yang et al., 2016; Chopik, 2017). However, the relative predictive power of social relatedness on physical health by different age groups is still largely understudied (Chopik, 2017). Moreover, the existing empirical evidence regarding the moderating effect of social relatedness on health is not straightforward. Some studies indicate that the protective power of perceived social support is stronger for older adults than for younger adults (Seeman et al., 2002; Yang et al., 2016). For instance, in a recent analysis that examined four representative samples of adults in the United States, Yang et al. (2016) found that the effect of social relationships on physiological functioning was particularly strong among those in adolescence and in late adulthood. In contrast, in another study utilizing one of the same data sets from Yang et al. (2016), Brooks et al. (2014) found the opposite pattern. In fact, the average support from family, friends, and spouses predicted higher levels of physiological dysregulation for older adults but lower levels for younger adults. Similarly, the empirical findings are inconsistent regarding the effect of loneliness in regulating age-related differences in health outcomes, with some studies supporting the role of loneliness in amplifying the age difference of physical health (Hawkley et al., 2006), while others show opposite or null findings (Hawkley et al., 2010; Whisman, 2010; Victor and Yang, 2012).

### Present study

Given the lack of evidence and inconsistency in the literature, the present research sets out to supplement findings in this area of research by conducting a cross-sectional study and a daily diary study. In Study 1, we utilized a cross-sectional design to examine how individuals' general feelings about social connectedness are associated with their physical health, with a particular focus on the possible moderating effect of age. In Study 2, we used a data set obtained from a daily diary study and tested whether daily experiences of loneliness could predict physical symptoms experienced during the study period after controlling for the initial physical symptoms. A daily diary study has additional advantages over traditional survey studies using retrospective assessments, in that it significantly reduces the time lapse between the experiences and the recall of those experiences (Reis and Gable, 2000; Bolger et al., 2003). Therefore, diary data can provide summary accounts of experiences with relatively fewer memory biases from retrospection over a long period. Thus, the findings from Study 1 and Study 2 can be expected to complement each other and to provide a comprehensive understanding of the relationship between social relatedness and health while considering age as an important boundary condition.

## Study 1

The aim of Study 1 was to examine whether the perception of social relatedness predicts physical health and whether age emerges as a moderator in this prediction. We focused on loneliness and perceived social support as capturing the absence and presence of social relatedness, respectively. Naturally, people who feel socially isolated and left out would be less likely to feel cared for and supported by their friends and family (Segrin and Passalacqua, 2010; Sarason, 2013). We expected that both the level of loneliness and perceived social support would be associated with physical health and that both associations would be qualified by age. Specifically, we expected that loneliness and perceived social support would be associated with physical health more strongly for older adults.

### Method

#### Participants and procedure

Participants were drawn from the Korean Adult Longitudinal Study, which was designed to be in parallel with the Midlife in the United States (MIDUS) and Midlife in Japan (MIDJA) projects. The purpose of the Korean Adult Longitudinal Study was to investigate social, psychological, and physiological development in adulthood and to reveal which of these developmental changes are associated with mental and physical well-being.

The sampling goal was 500 Seoul residents equally distributed across age groups (by decade from 20 to 60s), genders, and geographical areas in Seoul (Northeast, Southeast, Northwest, and Southwest). Participants were recruited via a research firm in Seoul, Korea. The participants were selected using random-digit dialing (RDD) of cell phone numbers with the age, gender, and residential information described above. Through the selection procedure, 519 Koreans (264 males, 255 females) aged 20–69 participated. The company oversampled initially with 732 adults as potential participants with the expectation of getting a response rate of ~70%. The participants answered a self-administered questionnaire distributed by the research company and were paid 10,000 Korean won (KRW) for the participation. The response rate was 72.4%, resulting in 530 participants who returned the questionnaire. An additional 11 participants were excluded from the final data due to low-quality responses. Of the remaining 519 participants, 148 individuals who also participated in the daily diary study (Study 2) were excluded from Study 1. Thus, the final sample for Study 1 was totaled 371 participants who participated exclusively in the Korean Adult Longitudinal Study. The mean age of the sample was 43.22 (*SD* = 14.61). The study was carried out in accordance with the recommendations of the Institutional Review Board at Seoul National University. All subjects gave written informed consent form approved by the Institutional Review Board at Seoul National University.

### Measures

#### Social relatedness

##### Loneliness

The Revised UCLA Loneliness Scale (Hughes et al., 2004) was used to assess the level of social isolation participants feel. Participants responded to the 3-items on a four-point scale ranging from 1 = *never* to 4 = *often* (e.g., “I feel left out”) (α = 0.82). The mean score was used for the analysis.

##### Perceived social support

Perceived social support was measured with the 12-item scale developed by Zimet et al. (1988). The participants were asked to rate on a 7-point scale (1 = not at all true, 7 = very true) the degree to which they believe they are getting support from three different sources, family, friends, and significant others (e.g., “There is a special person who is around when I am in need.”) (α = 0.92). The mean score was used for the analysis.

#### Physical health

##### Physical symptoms

Nine physical symptoms commonly used in MIDUS II, MIDUS III, and MIDJA were selected and the participants were to report how often they experienced each of the given symptoms in the past 30 days (1 = not at all, 2 = once a month, 3 = 2–3 times a month, 4 = once a week, 5 = 2–3 times a week, 6 = almost everyday). The symptoms were “headaches,” “backaches,” “sweating a lot,” “irritability,” “hot flushes or flashes,” “aches or stiffness in joints,” “trouble getting to sleep or staying asleep,” “leaking urine,” “pain or aches in extremities (arms/hands/legs/feet)” (α = 0.81). The responses were averaged across the nine items.

##### Chronic health conditions

The participants indicated whether they had either diagnosed or been treated in the past 12 months for each of 30 chronic health conditions commonly used in MIDUS II, MIDUS III, and MIDJA. The condition list included a broad range of conditions from “asthma, bronchitis or emphysema” and “tuberculosis” to “chronic sleeping problems” and “swallowing problems.” A total number of chronic health conditions were calculated and used for the analysis (See Appendix for the full list).

#### Covariates

##### Socio-demographic variables

Gender and Income Level were Used as Covariates. The income level was measured with eleven categories: (1) <1 million KRW, (2) 1–2 million KRW, (3) 2–3 million KRW, (4) 3–4 million KRW, (5) 4–5 million KRW, (6) 5–6 million KRW, (7) 6–7 million KRW, (8) 7–8 million KRW, (9) 8–9 million KRW, (10) 9–10 million KRW, and (11) >10 million KRW.

##### Health related behaviors

Health-related covariates included smoking status (0 = have no smoking experience, 1 = have a smoking experience) and amount of exercise (the average time spent on exercise on a daily basis measured in minutes).

#### Results and discussion

Table 1 shows descriptive statistics and correlations among measures. As predicted, indicators of social relatedness and health indicators were significantly correlated. Particularly, loneliness had positive correlations with physical symptoms and with the chronic health conditions. Consistent with loneliness, perceived social support had a significant negative correlation with physical symptoms and a marginal trend with chronic conditions.

**Table 1:** Descriptive statistics and correlations among measures in Study 1.

**Measure**	***M* (or %)**	***SD***	**1**	**2**	**3**	**4**	**5**	**6**	**7**	**8**
1. Gender	Male 52.3%	–	–							
2. Income	5.93	2.27	0.02	–						
3. Exercise	59.64	52.60	−0.09^†^	0.10^†^	–					
4. Smoking	Yes 38.5%	–	−0.65	−0.01	0.14^**^	–				
5. Perceived social support	5.32	0.78	0.01	0.16^**^	0.01	−0.11^*^	–			
6. Loneliness	1.69	0.64	−0.13^*^	−0.16^**^	−0.07	−0.20^***^	−0.44^***^	–		
7. Age	43.22	14.61	−0.04	−0.11^*^	0.15^**^	0.14^**^	−0.13^*^	−0.03	–	
8. Physical symptoms	1.79	0.77	0.07	−0.13^*^	−0.04	0.01	−0.12^*^	0.43^***^	0.10^†^	–
9. Chronic health conditions	0.75	1.53	−0.001	−0.15^*^	−0.11^*^	0.10^†^	−0.10^†^	0.30^***^	0.19^***^	0.58^***^

† p < 0.10;

* p < 0.05;

** p < 0.01;

*** *p < 0.001*.

We used hierarchical multiple regression to examine the association between social relatedness and physical health, and whether it is differentially associated depending on age. Following the recommendation of Aiken and West (1991), all continuous variables were centered. In Step 1, demographic variables of gender and household income level as well as health related habits such as average daily exercise time and current smoking status were included in order to control for their potential effects on physical symptoms. In Step 2, we included the main variables of interest, namely, the age of the participants and social relatedness (loneliness and perceived social support). The interaction term between social relatedness and age (loneliness x age and perceived social support × age, respectively) was entered in Step 3.

##### Physical symptoms and loneliness

**Figure 1:**
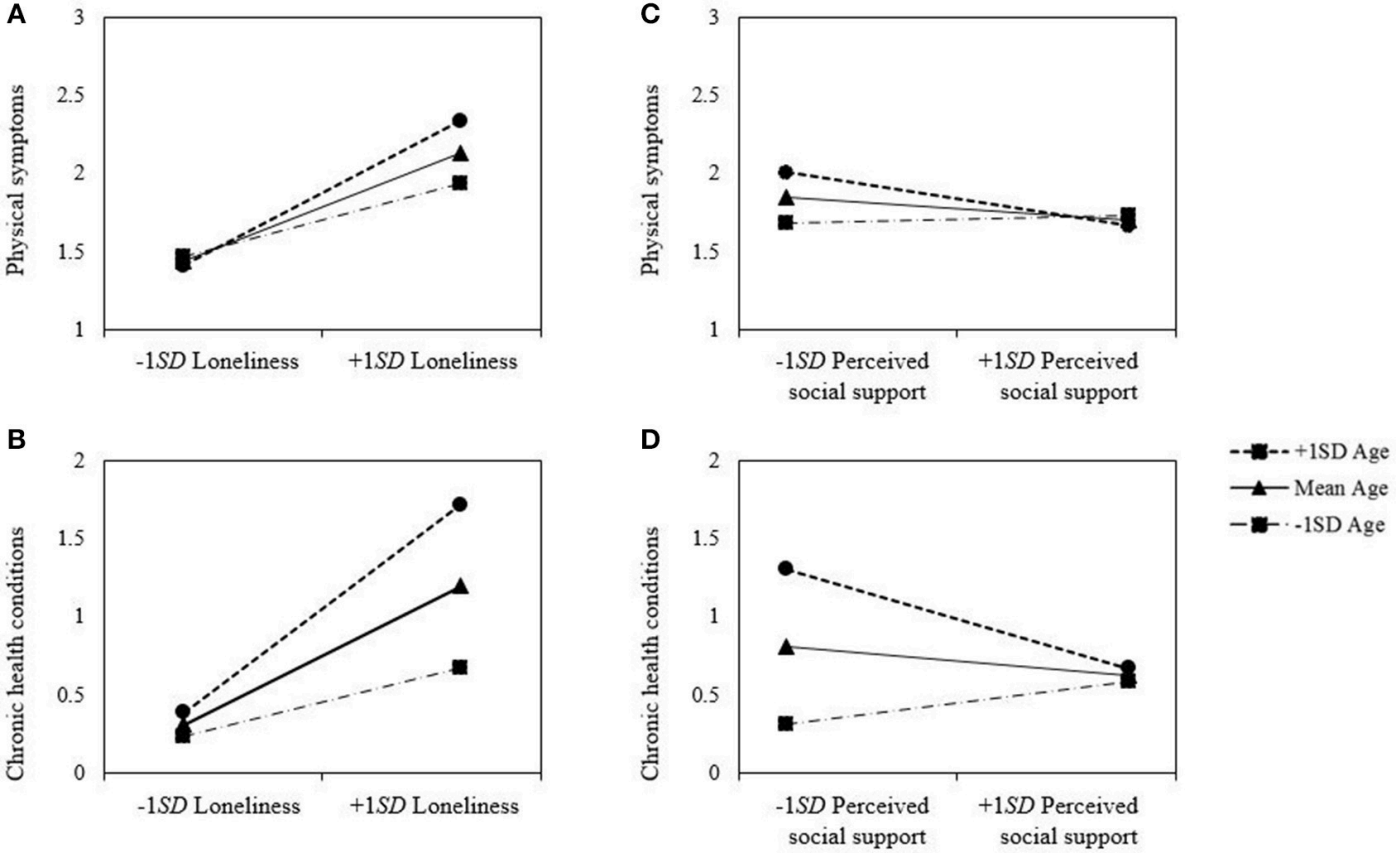
Moderation effect of age on the relation between **(A)** loneliness and physical symptoms, **(B)** loneliness and chronic health conditions, **(C)** perceived social support and physical symptoms, **(D)** perceived social support and health conditions in Study 1.

At the first step, the model predicting the physical symptoms by demographic variables and health related habits significantly accounted for the variance (*R*^2^ = 0.03, *p* = 0.034). Gender (β = 0.14, *p* = 0.041) and household income (β = −0.13, *p* = 0.016) were significantly predictive of physical symptoms. Compared with male participants, female participants reported greater physical symptoms. The participants of higher household income reported less physical symptoms. At the second step, introducing participants' loneliness and age led to a significant increase in the variance (Δ*R*^2^ = 0.19, *p* < 0.001). Both predictors, loneliness (β = 0.44, *p* < 0.001) and age (β = 0.11, *p* < 0.017) were significantly associated with greater physical symptoms, indicating that the participants who experienced greater loneliness suffered from higher levels of physical symptoms than those who experienced lower levels of loneliness and that older participants reported higher levels of physical symptoms than their younger counterparts. At the third step, the interaction between loneliness and age explained additional variance (Δ*R*^2^ = 0.02, *p* < 0.005). There was a significant age by perceived social support interaction effect (β = 0.14, *p* < 0.005). To test the moderation effect, further simple slope analyses were performed (Aiken and West, 1991). There was a significant positive association between loneliness and physical symptoms for the older adults (at 1 *SD* above the mean age), β = 0.60, *t* = 8.59, *p* < 0.001. There was also a significant, but relatively smaller, positive association between loneliness, and physical symptoms for the younger adults (at 1 *SD* below the mean age), β = 0.30, *t* = 4.49, *p* < 0.001. See Table 2 and Figure 1. These findings suggested that loneliness was more detrimental to physical health among older people than among younger people.

**Table 2:** Summary of hierarchical regression analysis for variable predicting physical symptoms and chronic health conditions in Study 1.

**Variables**	**Outcome**									
	**Physical symptoms**					**Chronic health conditions**				
	***B***	***SE B***	**95% CI**	**β**	**Δ*R*^2^**	***B***	***SE B***	**95% CI**	**β**	**Δ*R*^2^**
*Step 1*					0.03^*^					0.05^**^
Gender	0.21	0.10	[0.01, 0.42]	0.14^*^		0.32	0.20	[−0.08, 0.72]	0.11	
Income	−0.04	0.02	[−0.08, −0.01]	−0.13^*^		−0.09	0.04	[−0.16, −0.03]	−0.14^**^	
Exercise	0.000	0.001	[−0.002, 0.001]	−0.03		−0.003	0.002	[−0.01, 0.000]	−0.11^*^	
Smoking	0.16	0.11	[−0.05, 0.37]	0.10		0.55	0.21	[0.14, 0.97]	0.18^**^	
*Step 2*					0.19^***^					0.11^***^
Gender	0.20	0.09	[0.02, 0.39]	0.13^*^		0.27	0.19	[−0.11, 0.65]	0.09	
Income	−0.02	0.02	[−0.05, 0.02]	−0.05		−0.05	0.03	[−0.11, 0.02]	−0.07	
Exercise	0.000	0.001	[−0.001, 0.001]	−0.01		−0.003	0.001	[−0.01, 0.000]	−0.11^*^	
Smoking	−0.01	0.10	[−0.21, 0.18]	−0.01		0.27	0.20	[−0.14, 0.67]	0.09	
Loneliness	0.54	0.06	[0.42, 0.65]	0.44^***^		0.68	0.12	[0.45, 0.92]	0.28^***^	
Age	0.01	0.003	[0.001, 0.01]	0.11^*^		0.02	0.01	[0.01, 0.03]	0.20^***^	
*Step 3*					0.02^**^					0.02^**^
Gender	0.23	0.09	[0.05, 0.41]	0.15^*^		0.32	0.19	[−0.06, 0.70]	0.11^†^	
Income	−0.02	0.02	[−0.05, 0.02]	−0.04		−0.05	0.03	[−0.11, 0.02]	−0.07	
Exercise	0.000	0.001	[−0.001, 0.002]	0.02		−0.003	0.001	[−0.01, 0.000]	−0.09^†^	
Smoking	−0.01	0.10	[−0.20, 0.19]	−0.01		0.27	0.20	[−0.13, 0.67]	0.09	
Loneliness	0.54	0.06	[0.43, 0.66]	0.45^***^		0.70	0.12	[0.47, 0.93]	0.29^***^	
Age	0.01	0.003	[0.001, 0.01]	0.11^*^		0.02	0.01	[0.01, 0.03]	0.20^***^	
Loneliness × Age	0.01	0.004	[0.01, 0.02]	0.14^**^		0.02	0.01	[0.01, 0.04]	0.14^**^	

† p < 0.10;

* p < 0.05;

** p < 0.01;

*** *p < 0.001*.

##### Chronic health conditions and loneliness

The results replicated using the total number of chronic health conditions in the past 12 months as another measurement of physical health. The model predicting the chronic health conditions by demographic variables and health related habits significantly accounted for the variance in Step 1 (*R*^2^ = 0.05, *p* = 0.001). Household income (β = −0.14, *p* = 0.007), physical exercise (β = −0.11, *p* = 0.036), and smoking habit (β = 0.18, *p* = 0.009) were significantly associated with chronic health conditions. Participant who had higher household income, more physical exercise, and were non-smokers reported fewer chronic health conditions than those who had lower household income, less physical exercise, and were smokers. In Step 2, adding participants' loneliness and age accounted for extra variance (Δ*R*^2^ = 0.11, *p* < 0.001). Both loneliness (β = 0.28, *p* < 0.001) and age (β = 0.20, *p* < 0.001) had significant relationship with chronic health conditions. Participants who experienced less loneliness and were younger had fewer chronic health conditions. In Step 3, the two-way interaction between loneliness and age added significant increase in the variance (Δ*R*^2^ = 0.02, *p* = 0.004). A significant moderation effect of age on the relation between loneliness and chronic health conditions was found (β = 0.14, *p* = 0.004). Further simple slope tests revealed that the slope relating loneliness to chronic health conditions was significant for those older people, β = 0.44, *t* = 6.01, *p* < 0.001, while the slope was significant, but relatively lower, for those younger people, β = 0.15, *t* = 2.12, *p* = 0.035. As in the physical symptoms, the negative effect of loneliness on chronic health conditions became stronger with age.

##### Physical symptoms and perceived social support

As in the previous analyses for loneliness as an indicator of lack of social relatedness, the significant moderation effect of age was warranted using perceived social support (β = −0.13, *p* = 0.015). By conducting a series of simple slope analyses, the significant association between perceived social support and physical symptoms for the older participants was found, β = −0.22, *t* = −2.87, *p* < 0.005. In contrast, the relationship between social support and physical symptoms for the younger adults was not significant, β = 0.03, *t* = 0.44, *ns*. As the absence of social relatedness (i.e., loneliness) had greater effect on physical symptoms among older participants than among younger participants, the effect of perceived social support on physical symptoms was also stronger among older participants than among their younger counterparts.

##### Chronic conditions and perceived social support

As shown in Table 2, a consistent moderation effect of age was found using perceived social support, (β = −0.15, *p* < 0.005). Further simple slope analyses yielded the result consistent with the previous findings; there was a significant negative relationship between perceived social support and chronic health conditions for the older adults, β = −0.21, *t* = −2.77, *p* = 0.006, but not for the younger adults, β = 0.09, *t* = 1.31, *ns*. The results of the regression analyses are summarized in Table 3.

**Table 3:** Summary of hierarchical regression analysis for variable predicting physical symptoms and chronic health conditions in Study 1.

**Variables**	**Outcome**									
	**Physical symptoms**					**Chronic health conditions**				
	***B***	***SE B***	**95% CI**	**β**	**Δ*R*^2^**	***B***	***SE B***	**95% CI**	**β**	**Δ*R*^2^**
*Step 1*					0.03^*^					0.05^**^
Gender	0.21	0.10	[0.01, 0.42]	0.14^*^		0.32	0.20	[−0.08, 0.72]	0.11	
Income	−0.04	0.02	[−0.08, −0.01]	−0.13^*^		−0.09	0.04	[−0.16, −0.03]	−0.14^**^	
Exercise	0.000	0.001	[−0.002, 0.001]	−0.03		−0.003	0.002	[−0.01, 0.000]	−0.11^*^	
Smoking	0.16	0.11	[−0.05, 0.37]	0.10		0.55	0.21	[0.14, 0.97]	0.18^**^	
*Step 2*					0.01^†^					0.03^**^
Gender	0.19	0.10	[−0.02, 0.39]	0.12^†^		0.26	0.20	[−0.14, 0.66]	0.08	
Income	−0.04	0.02	[−0.07, 0.000]	−0.10^†^		−0.07	0.04	[−0.14, −0.01]	−0.11^*^	
Exercise	−0.001	0.001	[−0.002, 0.001]	−0.04		−0.004	0.001	[−0.01, −0.001]	−0.13^*^	
Smoking	0.12	0.11	[−0.10, 0.33]	0.07		0.43	0.21	[0.02, 0.84]	0.14^*^	
Perceived social support	−0.08	0.05	[−0.18, 0.02]	−0.08		−0.09	0.10	[−0.29, 0.11]	−0.05	
Age	0.004	0.003	[−0.001, 0.01]	0.08		0.02	0.01	[0.01, 0.03]	0.18^**^	
*Step 3*					0.02^*^					0.02^**^
Gender	0.21	0.10	[0.01, 0.41]	0.14^*^		0.31	0.20	[−0.09, 0.70]	0.10	
Income	−0.04	0.02	[−0.07, 0.000]	−0.10^†^		−0.07	0.03	[−0.14, −0.01]	−0.11^*^	
Exercise	0.000	0.001	[−0.002, 0.001]	−0.03		−0.004	0.001	[−0.01, −0.001]	−0.12^*^	
Smoking	0.12	0.11	[−0.09, 0.33]	0.07		0.44	0.21	[0.03, 0.85]	0.14^*^	
Perceived social support	−0.10	0.05	[−0.20, 0.01]	−0.10^†^		−0.12	0.10	[−0.31, 0.08]	−0.06	
Age	0.004	0.003	[−0.001, 0.01]	0.08		0.02	0.01	[0.01, 0.03]	0.18^**^	
Perceived social support × Age	−0.01	0.003	[−0.02, −0.002]	−0.13^*^		−0.02	0.01	[−0.03, −0.01]	−0.15^**^	

† p < 0.10;

* p < 0.05;

** p < 0.01;

*** *p < 0.001*.

Study 1 provided the evidence that the perception of social relatedness had positive association with physical health and this association was stronger for older adults. That is, higher levels of loneliness were associated with greater physical symptoms and this pattern was stronger for the older than for the younger. As for the perceived social support, the associations with physical symptoms were significant for the older only and not the younger. These results indicated that the lack/presence of social relatedness was more detrimental to/beneficial for the older adults compared to younger adults in health conditions. The consistent findings regarding the chronic health conditions confirmed that the moderating role of age was also present in the chronic health conditions as well as in the physical symptoms, which covered a relatively short-term period of time (i.e., in the past 1 month). In line with previous research, greater social support was related to positive effect on adaptation and recovery (Finlayson, 1976; Bromet and Moos, 1977; Wallston et al., 1983) and to fewer medical problems among elderly people (Hawkley et al., 2010).

Although the results of Study 1 provided initial evidence that the predictive power of social relatedness for physical health varies across age, given the cross-sectional design of the study, it is hard to rule out the possibility that the physical symptom might be an antecedent, rather than a consequence, of social relatedness. In order to address this limitation, we conducted a daily diary study and tested whether the experiences of the absence of social relatedness (i.e., loneliness) during a relatively short period (i.e., 13 days) could predict the physical symptoms experienced during that period, even after controlling for initial physical symptoms.

## Study 2

Study 2 was conducted to extend Study 1 in two ways. First, Study 2 attempted to replicate the results of Study 1 with daily measures of loneliness. We assessed the loneliness that participants experienced in the course a day via the daily diary method. Even though the method did not provide a perfect online measure of loneliness, it is closer to the participants' actual lived experience of loneliness in their daily lives and is less susceptible to emotional and cognitive biases compared to the retrospective measures of social relatedness used in Study 1 (Schwarz and Clore, 1983; Schkade and Kahneman, 1997; Schwarz and Strack, 1999; Robinson and Clore, 2002). Next, we examined whether the participants' daily experiences of loneliness over a relatively short period of time (i.e., 13 days) could predict the changes in physical health for the same period. To this end, the physical symptoms that participants experienced were measured before and after the 13-day daily diary study period. In other words, we tested whether loneliness was significantly associated with post-test physical symptoms after controlling for pre-test physical symptoms and whether its association was qualified by age.

### Method

#### Participants

A total of 407 individuals took part in a study called the Everyday Experience of Koreans, which included several surveys for multiple research projects, in return for monetary compensation of 50,000 Korean won. We used the daily diary part of the Everyday Experience of Koreans for Study 2. The participants were recruited through a research firm's panel on the basis of a stratified population sampling procedure in Seoul, Korea. Nineteen individuals were excluded because their answers were untrustworthy (e.g., they gave the same answers to all of the daily diary measures). Four were removed because their responses on the dependent variable (i.e., mean scores for physical symptoms over the course of 2 weeks) were more than 3 *SD* above the mean. Hence, 384 participants (50.8% females) were included in our analyses. Ages ranged from 31 to 69 (*M* = 50.04; *SD* = 10.92), with approximately equal numbers of participants from four age groups: 30s (*N* = 90), 40s (*N* = 96), 50s (*N* = 99), and 60s (*N* = 99). The study was carried out in accordance with the recommendations of the Institutional Review Board at Seoul National University. All subjects gave written informed consent using a form approved by the Institutional Review Board at Seoul National University.

#### Procedure

On the first day of the study, participants completed several online questionnaires, including surveys of demographic information (e.g., gender, age, marital status, level of education) and physical symptoms. From the second day of the study, participants filled out a daily diary for 13 subsequent days. Participants responded to daily diary measures via their own smartphones upon receiving a text message with a hyperlink that directed them to an online survey at 10:00 p.m. The participants were able to complete a daily diary measure at any time between 10:00 p.m. and 6:59 a.m. the next day. This step was taken in order to ensure adequate response rates (at the cost of some degree of memory bias) by allowing the participants to report loneliness of the previous day the next morning. The average number of daily diary responses per participants was 11.35 (*SD* = 1.76). A day after the daily diary study ended, participants were asked to complete a variety of questionnaires, including items about physical symptoms.

#### Measures

##### Pre/post-test physical symptoms

On the first day of the study, participants were asked to report whether they suffered from each of the nine somatic symptoms (yes/no) that were used in Study 1. The number of symptoms was used as an indicator of baseline physical health. A day after the daily diary study ended, the participants reported how much they had experienced the given symptoms in the past 2 weeks, on a 6-point Likert scale (1 = not at all, 2 = once in two weeks, 3 = 2–3 times in 2 weeks, 4 = 4–5 times in 2 weeks, 5 = once every 2 days, 6 = almost everyday). The participants were presented with the same nine somatic symptoms measured at baseline, with the additional category “other.” The post physical health scores were obtained by averaging the participants' ratings of all symptoms.

##### Daily loneliness

At each daily diary assessment, participants were asked to indicate the level of loneliness they felt on that day (“how lonely did you feel in the past 24 h?”) on a scale ranging from 0 (“not at all lonely”) to 10(“very lonely”). We calculated the scores of overall loneliness for each participant by averaging the levels of loneliness across the 13 days.

### Results and discussion

We examined whether the daily experience of loneliness over the course of 13 days had a detrimental effect on the participants' physical health and whether such a negative effect might be qualified by age—that is, whether the negative effect of loneliness on physical health might be more evident in older participants than in younger participants. To this end, we conducted multiple regression analysis in which post-test physical symptoms that participants reported the day after the daily diary study ended were treated as outcomes and the overall loneliness, age, and their interaction as predictors, after controlling for the pre-test physical symptoms participants reported the day before the daily diary study began. It is common to use multilevel linear modeling for the analyses of daily diary data, since the data have a hierarchical structure: the daily level (level 1) and the person level (level 2). In our study, however, multilevel linear modeling was not appropriate, since the outcome (i.e., post-physical symptom) is measured at level 2 (i.e., the person level), not level 1 (i.e., the daily level). Furthermore, whereas the new statistical methods such as the latent variable two-step approach (Croon and van Veldhoven, 2007) and the Full Information Maximum Likelihood latent variable technique (Lüdtke et al., 2008) were introduced for multilevel data with group-level outcomes, a recent simulation study showed that OLS analysis of the group mean not only provides low bias in estimations and good statistical power compared to the new techniques but also is free of convergence problems (Kromrey and Foster-Johnson, 2015). Hence, we aggregated loneliness scores measured at the daily level so that the loneliness and person-level variables can be included in a single level of analysis.

The correlation analyses showed that overall loneliness was positively related to post-test physical symptoms. Gender and age were not correlated with post-test physical symptom. Means, standard deviations, and intercorrelations for variables are shown in Table 4. Subsequently, we conducted three-step hierarchical regression. Covariates such as gender and pre-test physical symptom were entered in the first step. Overall loneliness and age were entered in the second step, and interaction between the loneliness and age were entered in the third step. All variables in the interaction terms were mean-centered.

**Table 4:** Descriptive statistics and correlations among measures in Study 2.

**Measure**	***M***	***SD***	**1**	**2**	**3**	**4**	**5**
1. Gender	0.51	0.50	–				
2. Pre-test physical symptom	1.40	1.05	0.08	–			
3. Age	50.04	10.92	0.003	−0.14^***^	–		
4. Loneliness	4.47	1.86	−0.12^***^	0.20^***^	−0.16^***^	–	
5. Post-test physical symptom	2.13	0.82	0.11	0.52^***^	0.003	0.33^***^	–

† p < 0.10;

* p < 0.05;

** p < 0.01;

*** *p < 0.001*.

As presented in Table 5, the results revealed that pre-test physical symptoms (β = 0.512, *p* < 0.001) in the first step (*R*^2^ = 0.27, *p* < 0.001) were significantly associated with post-test physical symptoms, but gender was not (β = 0.070, *p* = 0.111). As expected, there was a strong association between pre- and post-test physical symptoms. Both overall loneliness (β = 0.268, *p* < 0.001) and age (β = 0.113, *p* = 0.008) in the second step (Δ*R*^2^ = 0.072, *p* < 0.001) positively predicted post-test physical symptoms, indicating that participants who experienced more loneliness during the daily-diary study experienced more physical symptoms than did those who experienced less loneliness, after controlling for baseline physical symptoms and that older participants suffered from more physical symptom than did younger participants.

**Table 5:** Summary of hierarchical regression analysis for variable predicting post physical symptom in Study 2.

**Variables**	**Post-physical symptom**				
	***B***	***SE B***	**95% CI**	**β**	**Δ*R*^2^**
*Step 1*					0.27^***^
Gender	0.11	0.07	[0.02, 0.26]	0.07	
Pre-physical symptom	0.40	0.03	[0.33, 0.47]	0.51^***^	
*Step 2*					0.07^***^
Gender	0.17	0.07	[0.04, 0.31]	0.11^*^	
Pre-physical symptom	0.37	0.03	[0.30, 0.43]	0.47^***^	
Loneliness	0.12	0.02	[0.08, 0.16]	0.27^***^	
Age	0.01	0.003	[0.002, 0.02]	0.11^**^	
*Step 3*					0.01^*^
Gender	0.19	0.11	[0.05, 0.32]	0.11^**^	
Pre-physical symptom	0.37	0.03	[0.30, 0.43]	0.47^***^	
Loneliness	0.12	0.02	[0.08, 0.16]	0.27^***^	
Age	0.01	0.003	[0.002, 0.02]	0.12^**^	
Loneliness × Age	0.004	0.002	[0.001, 0.007]	0.09^*^	

† p < 0.10;

* p < 0.05;

** p < 0.01;

*** *p < 0.001*.

More importantly, the main effect of loneliness on the post-test physical symptoms was significantly qualified by age (β = 0.094, *p* = 0.025) in the third step (Δ*R*^2^ = 0.009, *p* = 0.025). We decomposed the interaction by examining how loneliness influenced physical symptoms in older (−1 *SD*) and younger (+1 *SD*) adults. Simple slope analysis showed that the association between loneliness and post-test physical symptoms was stronger among older adults, β = 0.368, *t* = 5.95, *p* < 0.001, compared to among younger adults, β = 0.171, *t* = 2.82, *p* = 0.005, suggesting that the detrimental effect of loneliness becomes stronger with age. The findings of Study 2 indicated that the daily experiences of loneliness over a 13-day period were negatively associated with physical health and that this negative association was more evident for older participants.

## General discussion

The present research provided consistent findings across Study 1 and Study 2, indicating that social relatedness (i.e., higher levels of loneliness and lower levels of perceived social support) is detrimental to physical health and that this effect is stronger for older adults compared to younger adults. In Study 1, using loneliness and perceived social support as two indices of social relatedness, we showed that subjective feelings of social connectedness were associated with physical health for older adults to a greater degree than for younger adults. Specifically, higher loneliness was associated with worse physical symptoms and chronic conditions, and this pattern was stronger for older adults than for younger adults. Regarding perceived social support, the associations with physical symptoms and chronic symptoms were significant for older adults but not for younger adults. Interestingly, although it did not reach statistical significance, the association between perceived social support and chronic conditions was positive for younger adults. It would be interesting to explore this pattern further in future research. There is empirical evidence of an adverse relationship between one's well-being and social support (Williams et al., 2017), whereby individuals are excessively dependent on and preoccupied with relationships such that the balance between autonomy and interdependency is disrupted (Blatt, 2008). Study 2 further showed that when controlling for the initial symptoms, the loneliness experienced over the 13 days amplified the age difference in physical symptoms afterwards. Together, these findings provide support for the importance of considering age differences in examining the benefits that social relatedness can bring to health.

It is worth noting that this study is one of the first to examine the relationship between the social relatedness and physical health of Korean adults, especially those of a wide age range. Not only were previous studies on the link between social relatedness and physical health across different age ranges conducted mostly in Western countries, but also those that were conducted among Koreans focused primarily on elderly populations (Kim et al., 2005; Wong et al., 2007; Shin et al., 2008). By including participants of a wide age range, the present study was able to identify the conditions under which the perception of social connection was particularly influential to one's health.

Our attempt not only allows us to expand this research to a new cultural context (i.e., Korea) but also provides a unique contribution to the literature examining the role of cultural context in understanding health processes. In recent years, researchers have begun to consider cultural background as an important moderators in explaining the seemingly inconsistent associations between social relationships and well-being (Kim et al., 2008; Uchida et al., 2008; Park et al., 2013; Campos and Kim, 2017; Ishii et al., 2017). Given the strong interdependent and collectivistic cultural norms in Korea (Triandis, 1993; Oyserman et al., 2002), a sense of belongingness and supportive social relationships may be particularly critical for one's well-being in this cultural context (Kitayama et al., 2006; Uchida and Kitayama, 2009). Also, the fact that older (vs. younger) adults' health is more influenced by the perception of social connectedness may indicate that the interdependent cultural values are more pertinent to older than to younger adults in the Korean context. It would be a worthwhile task for future research to explore whether cultural values have any role in amplifying the impact of the perception of social connectedness.

There are several limitations of the present study that are worthy of consideration. First, our measure of health relied on self-reports, which are more closely associated with subjective evaluations of one's health status than objectively measured biomarkers. Although the evaluation of chronic health conditions was objective in the sense that these conditions were either diagnosed or treated by health professionals, such measures are nonetheless less accurate than biomarker measurements. Future work should also employ objective measures of health indicators. Second, we compared the relative strength of the association between social relatedness and physical health across different age range. Hence, it is hard to determine whether the observed difference in the effect of social relatedness on health across age is due to aging effects, cohort effects, or a combination of the two. Longitudinal studies using several methods, such as self-report measures, daily diary measures and experience sampling measures, are needed in order to illuminate this issue. In addition, although we used the daily diary method for Study 2, we did not conduct a within-person analysis of the relationship between loneliness and physical well-being, which would have shed further light on the potential fluctuations of the relationship between these two factors over the two-week period.

Healthy aging is one of the major tasks that individuals face in a time when life expectancy will soon exceed 90 years for the first time in human history (Kontis et al., 2017). In fact, the rate of the increase in the national life expectancy does not seem to be slowing down (Oeppen and Vaupel, 2002). South Korea, in particular, along with some Western countries, is one of the top performers in the rise in life expectancy. Nearly 60% of the South Korean girls born in 2030 will be likely to have a life expectancy of 90 years (Kontis et al., 2017). As individuals spend more years in later life, maintaining healthy physical status has never been more critical in determining one's quality of life, and loneliness is indeed a troubling challenge of getting old for most lay people (Pew Research Center, 2009). According to the findings of the present research, the consequences of loneliness among the older population are not unreasonable. Studies conducted in the West document that up to 40% of people aged 60 or older experience loneliness (Dickens et al., 2011). In the process of rapid societal change in recent years in Korea, there has been as much as a fourteen-fold increase in the divorce rate among the elderly compared to 20 years ago (Statistics Korea, 2014). Considering that spousal support is a major source of social support (Dehle et al., 2001), social isolation among older individuals has thus become an important social problem now more than ever (Jung, 2017). The findings of the present research call policymakers' attention to the need for intervention programs providing social support in a group format targeting the older population such as group discussions, group physical activities, and network-building programs at various sites including nurseries, clinics, and hospitals (See Dickens et al., 2011 for a review).

## Author contributions

EC is the first author on this paper–she led study design and implementation, analyses, and manuscript preparation; YK, and ML assisted with study design, implementation, and manuscript preparation; JC is the corresponding author on this paper–he led study design, implementation, analyses, and manuscript preparation; IC is the senior author on this paper, he oversaw study design, implementation, and analyses.

### Conflict of interest statement

The authors declare that the research was conducted in the absence of any commercial or financial relationships that could be construed as a potential conflict of interest.

## Appendix

Full list of items used for chronic health conditions in Study 1
In the past 12 months, have you experienced or been treated for any of the following?
a. Asthma, bronchitis, or emphysema
b. Tuberculosis
c. Other lung problems
d. Arthritis, rheumatism, or other bone or joint diseases
e. Sciatica, lumbago, or recurring backache
f. Persistent skin trouble (e.g., eczema)
g. Thyroid disease
h. Hay fever
i. Recurring stomach trouble, indigestion, or diarrhea
j. Urinary or bladder problems
k. Being constipated all or most of the time
l. Gall bladder trouble
m. Persistent foot trouble (e.g., bunions, ingrown toenails)
n. Trouble with varicose veins requiring medical treatment
o. AIDS or HIV infection
p. Lupus or other autoimmune disorders
q. Persistent trouble with your gums or mouth
r. Persistent trouble with your teeth
s. High blood pressure or hypertension
t. Anxiety, depression, or some other emotional disorder
u. Alcohol or drug problems
v. Migraine headaches
w. Chronic sleeping problems
x. Diabetes or high blood sugar
y. Multiple sclerosis, epilepsy, or other neurological disorders
z. Stroke
aa. Ulcer
bb. Hernia or rupture
cc. Piles or hemorrhoids
dd. Swallowing Problem
